# Lamotrigine-Induced Lupus With Aseptic Meningitis and Hemophagocytic Lymphohistiocytosis

**DOI:** 10.7759/cureus.29629

**Published:** 2022-09-26

**Authors:** Dena H Tran, Kory S Jaggon, Jamal Mikdashi, Robert D Chow, Avelino C Verceles, Aseem Sood

**Affiliations:** 1 Internal Medicine, University of Maryland Medical Center Midtown Campus, Baltimore, USA; 2 Rheumatology, University of Maryland School of Medicine, Baltimore, USA; 3 Internal Medicine, University of Maryland School of Medicine, Baltimore, USA; 4 Pulmonary and Critical Care Medicine, University of Maryland School of Medicine, Baltimore, USA

**Keywords:** hemophagocytic lymphohistiocytosis (hlh), aseptic meningitis, lamotrigine, systemic lupus erythematosus, autoimmune disorder

## Abstract

Hemophagocytic lymphohistiocytosis (HLH) is a life-threatening syndrome characterized by disordered immune activation resulting in cytokine storm and inflammation. We present a 27-year-old woman who had a fever and diffuse rash after recently starting lamotrigine. She developed meningismus and polyarthralgia. Laboratory results revealed cytopenia, elevated serum aminotransferases, hypofibrinogenemia and elevated ferritin. Cerebrospinal fluid analysis suggested aseptic meningitis. Antinuclear antibody and rheumatoid factor serologies were positive, complement levels of C3 were decreased, and antihistone antibody was negative. A bone marrow biopsy demonstrated hemophagocytic macrophages and the diagnosis of HLH was made. The patient was empirically started on high-dose intravenous dexamethasone following which both her mental status and laboratory indices markedly improved. Lamotrigine has been shown to induce lupus-like syndrome, aseptic meningitis, and HLH, but not concomitantly. Our patient was recently started on lamotrigine, likely inducing her underlying undiagnosed lupus, in addition to, resulting in aseptic meningitis and a cytokine storm leading to HLH.

## Introduction

Hemophagocytic lymphohistiocytosis (HLH) is a potentially life-threatening syndrome characterized by disordered immune activation, resulting in cytokine storm and inflammation. HLH is either classified as primary or secondary, with the primary form related to genetic mutations resulting in abnormal immune function [1,2], and the secondary (reactive form) occurring in response to antigen challenges such as an autoimmune disease, malignancy, or infection. The primary form is more common in the pediatric population and is well-studied of the two types, with data being sparse on the secondary or the adult form. Secondary HLH occurring in the setting of an autoimmune disease can be referred to as macrophage activation syndrome (MAS). Adult type HLH is relatively uncommon, with rates of approximately 1 per 800,000 [3]. We present a case of a young woman who received lamotrigine, likely inducing her underlying undiagnosed lupus in addition to aseptic meningitis and hemophagocytic lymphohistiocytosis. 

## Case presentation

A 27-year-old woman presented to the emergency department (ED) with fever, generalized diffuse rash, arthralgias and confusion after recently starting lamotrigine two weeks prior to presentation. Her medical history was significant for childhood seizure and she was recently diagnosed with partial complex seizure with temporal lobe epilepsy by an electroencephalogram (EEG) and started on lamotrigine 25 milligrams (mg) by a neurologist. A few days later she developed arthralgias involving the shoulders, wrists, knees, and ankles symmetrically, as well as neck pain, nausea and vomiting. Two days later, she noted intermittent fever and chills (maximum temperature 103.8 degrees Fahrenheit) accompanied by headache and painful neck lymphadenopathy. She also noticed a generalized, diffuse, maculopapular, nonpruritic rash involving the entire skin surface while showering. There was associated neck stiffness and photophobia. She presented to the ED for concern that her symptoms were related to lamotrigine, and she discontinued that medication the day before presentation. She had a recent history of travel to Japan three months prior. There was no history of alcohol, illicit substance, or tobacco use.

**Figure 1:**
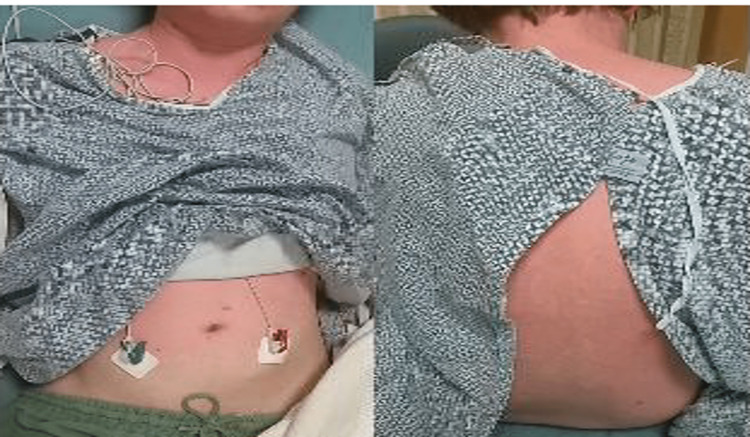
Diffuse erythematous rash after the initiation of lamotrigine.

Upon presentation to the ED, vitals were significant for temperature 101.8 degrees Fahrenheit, heart rate 108 beats/minute, and blood pressure 96/62 mmHg. Physical examination was remarkable for symmetrical cervical lymphadenopathy and a maculopapular, erythematous, blanching, and confluent rash involving the upper extremities, torso, and proximal lower extremities to the knees (Figure 1). The patient was alert and oriented to time, person and place but would later become confused and lethargic. No neck stiffness was appreciated.

Laboratory data was significant for neutropenia, thrombocytopenia, transaminitis, hypofibrinogenemia, elevated C-reactive protein and elevated sedimentation rate (Table 1).

**Table 1:** Laboratory Results Anti-SSA antibody: anti-Sjogren's syndrome-related antigen A autoantibodies

Laboratory Results	Result	Reference
White Blood Cell	3.4 K/mcL	4.8-10.9 K/mcL
Lymphocytes	0.3 K/mcL	1.0-4.8 K/mcL
Monocytes	0.0 K/mcL	0.3-0.7 K/mcL
Neutrophils	3.1 K/mcL	1.8-77 K/mcL
Hemoglobin	12.4 g/dL	12-16 g/dL
Platelets	80 K/mcL	153-367 K/mcL
International Normalized Ratio	1.87	
Prothrombin Time	22.0 sec	12.1-14.9 sec
Partial Thromboplastin Time	79.1 sec	23.9-41.3 sec
Fibrinogen	162 mg/dL	194-475 mg/dL
Serum glucose	101 mg/dL	74-106 mg/dL
Total protein	84. g/dL	6.3-8.2 g/dL
Albumin	3.8 g/dL	3.5-5.0 g/dL
Aspartate Aminotransferase	225 u/L	14-59 u/L
Alanine Aminotransferase	120 u/L	9-52 u/L
Alkaline phosphatase	214 u/L	38-126 u/L
Total bilirubin	1.2 mg/dL	0.2-1.3 mg/dL
C-reactive protein	8.90 mg/dL	0.00-0.99 mg/dL
Sedimentation rate	25 mm/hour	0-20 mm/hour
Antinuclear antibody	Positive	
Rheumatoid Factor	Positive	
Anti-SSA antibody	>0.8	0-0.9
Antihistone antibody	0.7	0-0.9
Complement C3 level	78 mg/dL	88-165 mg/dL
Complement C4 level	19 mg/dL	14-44 mg/dL
Cerebrospinal Fluid Analysis Results		
White Blood Cell	520	0-5
Protein	290	12-60 mg/dL
Glucose	76	40-70 mg/dL
Polymorphonuclear leukocytes	93%	

Due to the initial concern for sepsis and meningitis, the patient was empirically started on broad-spectrum antibiotics, including ceftriaxone, vancomycin, acyclovir, and doxycycline. The latter was added because the patient resided in an area endemic for Lyme disease. Cerebrospinal fluid (CSF) from a lumbar puncture revealed an opening pressure 28 mm H_2_O, WBC 520, 93% polymorphonuclear leukocytes, protein 290 mg/dL, and glucose 76 mg/dL consistent with aseptic meningitis (Table 1). Cytomegalovirus polymerase chain reaction, Lyme disease antibodies, peripheral smears for Babesia, *Ehrlichia* antibodies, respiratory viral panel including influenza, Parainfluenza and Adenovirus, Rocky Mountain Spotted Fever antibodies on CSF, *Bartonella henselae *and *Bartonella quintana* antibodies were all negative. Lactate, blood cultures and human immunodeficiency virus (HIV) testing were negative. All empiric antibiotics were subsequently discontinued given a lack of improvement and the negative studies above.

Further laboratory investigation revealed positive antinuclear antibody (ANA), positive rheumatoid factor (RF), positive anti-SSA antibody (Rho), negative antihistone antibody, normal anti-double stranded DNA, and low complement C3 level. Ferritin was significantly elevated at 18,154 ng/mL (upper limit of normal 137 ng/mL). Right upper quadrant ultrasound showed a prominent liver at 16.7 cm. Magnetic resonance imaging (MRI) brain showed no intracranial pathology but did reveal enlarged cervical lymph nodes with evidence of cystic degeneration or necrosis. Additionally, the patient had pleural effusions.

**Figure 2:**
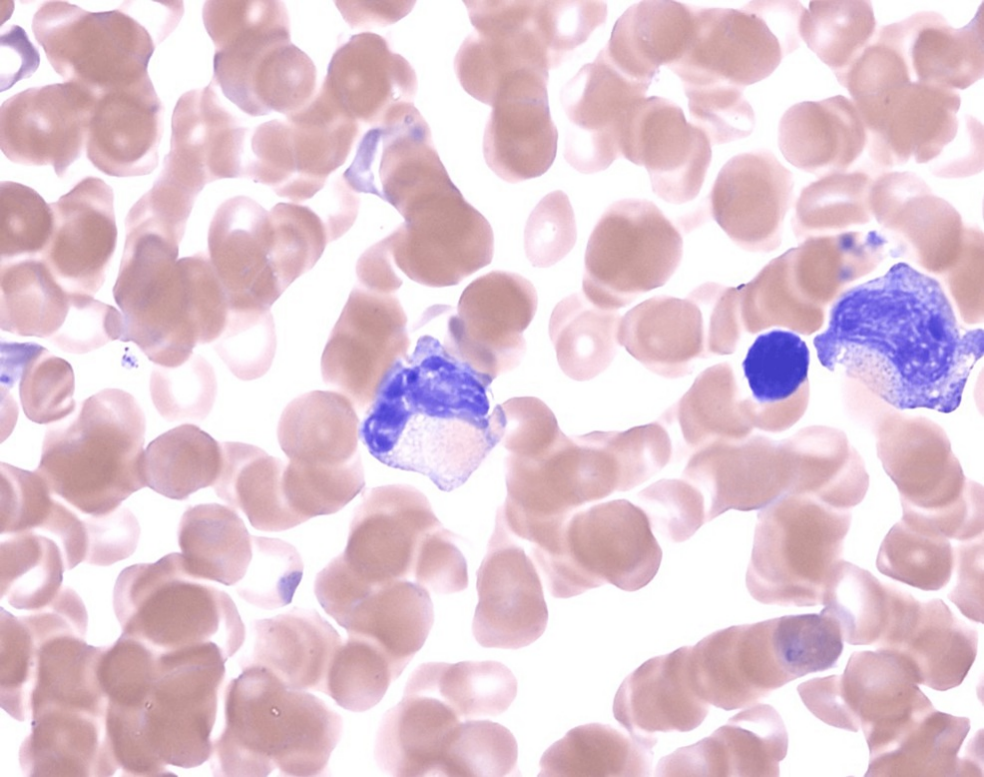
Hemophagocytic macrophages identified on bone marrow biopsy.

After infectious etiologies were deemed less likely, differential diagnosis focused on the recently prescribed lamotrigine, and included Drug Rash with Eosinophilia and Systemic Symptoms (DRESS), though the patient did not have peripheral eosinophilia. Given the elevated ANA, RF, low complement C3 level and elevated ferritin, there was concern for an underlying autoimmune disease such as lupus with concomitant hemophagocytic lymphohistiocytosis. She was empirically started on methylprednisolone 500 mg daily for two days and was switched to dexamethasone 17 mg daily (10 mg/m2 BSA) in accordance with the HLH-94 treatment protocol [4]. She received dexamethasone for a total of seven days. Within one to two days of starting this therapy, the patient’s mental status and laboratory parameters improved. The patient had a bone marrow biopsy on hospital day 5, three days into her course of steroids that showed histiocytes with phagocytosed erythroid precursors, red cells, platelets and nuclear debris (Figure 2).

This result, along with the clinical features above, was consistent with the diagnosis of HLH, as she had fulfilled the HLH diagnostic criteria [5]. She was discharged home to complete an oral dexamethasone taper over the course of six weeks, as recommended in the HLH-94 guidelines. Notably, she did have a positive Epstein-Barr virus (EBV)DNA in whole blood, which raised concern for her symptoms being related to EBV infection. EBV has been reported to be a trigger for HLH [6].

## Discussion

Adult hemophagocytic lymphohistiocytosis (HLH) remains a diagnostic challenge. HLH is an uncommon entity regarding the incidence when compared to childhood HLH. Incidence rates regarding the pediatric form vary widely, with estimates ranging between 1 to 225 per 300,000 live births [7]. One nationwide survey in Japan reported an incidence of both adult and pediatric cases at 1 per 800,000 [3], while another study estimated the incidence to be as high as 1 in every 2,000 adult admissions at tertiary medical centers [8]. There has been a steady increase in the number of reports about HLH published between 1979 and 2021, with the majority being single-center retrospective studies. Whether this is reflective of an increased incidence of the disease over time or increased awareness is unclear [1].

Our patient was started on lamotrigine for a new diagnosis of epilepsy, which likely triggered an underlying undiagnosed lupus autoimmune process, in addition to aseptic meningitis and leading to activation of cytokine storm resulting in hemophagocytic lymphohistiocytosis. Lamotrigine has been shown to induce lupus-like syndrome [9], been associated with aseptic meningitis [10,11], and hemophagocytic lymphohistiocytosis [12,13], however, there have been few reports showing these associations occurring concomitantly. Our patient developed all of these associations, likely triggered by the use of lamotrigine.

Per the European League Against Rheumatism (EULAR) and the American College of Rheumatology (ACR), the 2012 Systemic Lupus International Collaborating Clinics (SLICC) criteria require four or more criteria to be met, with at least one criterion being clinical and one laboratory [14]. Our patient presented with neutropenia, thrombocytopenia, low complement C3 level, arthralgia and pleural effusions, in addition to testing positive for ANA and RF serology and would meet the criteria for the diagnosis of systemic lupus erythematosus (SLE). As previously stated, this may have been related to lamotrigine though her antihistone antibody (55-92% sensitivity and 69-82% specificity for lupus) was negative [15,16].

When HLH occurs in the context of an autoimmune disease, it is termed macrophage activation syndrome (MAS). MAS is most commonly associated with systemic juvenile idiopathic arthritis (sJIA) and occurs in approximately 7-13% of patients with sJIA [17]. MAS can also occur with adult-onset Still’s diseases, SLE, Kawasaki disease, juvenile dermatomyositis, antiphospholipid syndrome and mixed connective tissue disease [17,18]. Literature regarding MAS is limited, with a recent review identifying only seven cases published in the literature between 2007-2017 [18]. The rarity of this disease combined with the simultaneous appearance of symptoms related to the underlying rheumatologic disease can make diagnosis challenging [18]. Additionally, there have been eight reported cases thus far documenting the association of lamotrigine inducing HLH [12]. Although the finding of hemophagocytosis on bone marrow (BM) biopsy is suggestive, this finding is only present in about 60% of patients [19].

In 2016, the American College of Rheumatology published classification criteria to assist with the diagnosis of MAS occurring in JIA [5] that includes ferritin >684 ng/ml and any two of: thrombocytopenia, elevated Aspartate aminotransferase (AST), elevated triglycerides and low fibrinogen. Our patient met all of these criteria. Prior to that time, the diagnosis was largely based on the HLH-2004 criteria [5]. The patient also fulfilled the full HLH-2004 criteria, meeting five out of nine criteria and also having bone marrow findings of hemophagocytosis. It is important to note, however, that the 2016 criteria were developed for the pediatric population with sJIA rather than the adult-onset form of the disease.

Regarding the treatment of adult patients with MAS, data are extremely limited, with no clinical evidence supporting the commonly used regimens. Treatment approaches are often extrapolated from case series and case reports. Regarding adult onset HLH, there have also been no prospective trials to date with most treatment regimens based on the HLH-94 study in children <16 years old [7]. The typical regimen consists of induction with dexamethasone and etoposide, followed by a calcineurin inhibitor such as cyclosporine or tacrolimus. Those with persistent disease often require hematopoietic cell transplantation. Without treatment, HLH is a deadly illness, with mortality ranging from 52%-75% [20]. This contrasts with MAS which, while still carrying a high mortality at 8-22%, is able to be controlled in up to half of the cases with steroids alone [17]. This was in keeping with our patient’s response to therapy, improving in all laboratory parameters and clinically without the need for additional immunosuppressive drugs. The patient continues to do well to date since her HLH diagnosis six years prior.

## Conclusions

Hemophagocytic lymphohistiocytosis (HLH) is a rare and potentially fatal complication that may be a result of certain medications and autoimmune diseases. Our patient was recently started on lamotrigine, likely inducing her underlying undiagnosed lupus, in addition to, resulting in aseptic meningitis and a cytokine storm leading to HLH concomitantly. HLH should be considered as a differential diagnosis in patients with persistent fevers, cytopenia, significantly elevated ferritin, and/or hypofibrinogenemia. Patients diagnosed with HLH should be treated immediately with high-dose steroids due to high mortality rates.
